# Déterminants Socio-Anthropologiques de la Prévalence Élevée des Fistules Obstétricales en Guinée

**DOI:** 10.48327/mtsibulletin.n1.2021.68

**Published:** 2021-03-15

**Authors:** A. Diallo, I.S. Baldé, G. Loua, N. Diakité, O. Baldé, F.B. Diallo, I.T. Diallo, A. II. Sow, M. Diallo

**Affiliations:** 1 Service de gynécologie-obstétrique de l'Hôpital national Ignace Deen, Conakry, Guinée; 2 Organisation panafricaine de lutte pour la santé (OPALS); 3 Programme national de lutte contre le paludisme en Guinée (PNLP); 4 Service de gynécologie-obstétrique de l'Hôpital national Donka, Conakry, Guinée

**Keywords:** Fistules obstétricales, Sorcellerie, Djinns, Rupture d'interdits, Volonté divine, Excision, Deuxième excision, Abstinence de fin de la grossesse, Mariages précoces, Travail dystocique, Dystocie, Accouchement à domicile, Déterminants socio-anthropologiques, Fistule obstétricale, Services de santé, Malinké, Peul, Soussou, Labé, Dubréka, Kissidougou, Guinée, Afrique subsaharienne, Obstetric fistula, Witchcraft, Djinns, Breaking of taboos, Divine will, Excision, Second excision, Abstinence at the end of pregnancy, Early marriage, Dystocic work, Obstructed labour, Home delivery, Socio-anthropological determinants, Obstetric fistulas, Health services, Malinké, Peul, Soussou, Labé, Dubréka, Kissidougou, Guinea, Sub-Saharan Africa

## Abstract

**Objectifs:**

L'objectif de cette étude était d'analyser les déterminants socio-anthropologiques (représentations, croyances, pratiques et perceptions des services de santé) de la prévalence élevée des fistules obstétricales en Guinée.

**Patientes et méthodes:**

Il s'agissait d'une étude qualitative d'une durée d'un mois, du 15 janvier au 15 février 2018, réalisée dans trois centres de santé (un urbain et deux ruraux). Elle a concerné les mères biologiques qui sont venues faire vacciner leurs enfants. Les données ont été collectées par entretien individuel semi-directif.

**Résultats:**

Aucune des 42 répondantes n'a pas pu faire le lien entre la survenue des fistules obstétricales et l'accouchement dystocique. Dans les trois sites de l'étude, on pensait que l'accouchement dystocique et la fistule obstétricale ont une origine mystique. À Kissidougou, les répondantes pensaient que les accouchements dystociques et les fistules obstétricales sont causées, soit par la sorcellerie, *soubaya* en malinké ou un mauvais sort *korte* en malinké, jeté par une personne ennemie, ou le mauvais comportement de la parturiente, c'est-à-dire lorsqu'elle pratique l'adultère ou si elle a un comportement irrespectueux vis-à-vis des aînés. À Dubréka, les répondantes liaient la survenue de la dystocie et de la fistule obstétricale à la sorcellerie, *koromikhi* en soussou À Labé, certaines répondantes pensaient que l'accouchement dystocique et la fistule obstétricale sont dus à une punition divine, en peul *lette Allah*, lorsque la femme ne respecte pas son mari ou si elle a contracté la grossesse hors mariage. D'autres pensaient que l'accouchement est difficile à cause de l'étroitesse de la voie d'accouchement, en peul *lawol ngol no faadhi*, chez les parturientes qui n'ont pas de rapports sexuels pendant la grossesse ou encore lorsque la femme n'a pas été proprement excisée, en peul *o suuwaaki laabhi*, c'est-à-dire lorsqu'on a laissé une partie du clitoris en place lors de l'excision. La majorité des répondantes avait une mauvaise perception des services de santé (présence de personnel de sexe masculin, non-respect de l'intimité, mauvaise hygiène, maltraitance).

**Plusieurs pratiques culturelles:**

(mariages précoces, mutilations génitales féminines, interdits alimentaires, accouchements à domicile) favorisent également la survenue des fistules obstétricales.

**Conclusion:**

Les croyances et les pratiques culturelles en matière d'accouchement des répondantes limitent leur fréquentation des maternités pour accoucher et favorisent la survenue des fistules obstétricales.

## Abstract (en)

**Objective:**

The purpose of this study was to analyse the socio-anthropological determinants (representations, beliefs, practices and perceptions of health services) of the high prevalence of obstetric fistulas in Guinea.

**Patients and methods:**

From January 15 to February 15, 2018, we carried out a qualitative study in three health centres (one urban and two rural). The study focused on biological mothers who attended the centres to get vaccinated their children. The data were collected by semi-structured individual interview.

**Results:**

None of the 42 respondents could link the occurrence of obstetric fistulas and obstructed labour. Participants at all three study sites believed that obstructed labour and obstetric fistula have a mystical origin. In Kissidougou, the respondents thought that obstructed labour and obstetric fistulas are caused either by *soubaya*, sorcery in Malinké or the evil spell *korte* in Malinké cast by an enemy, or the bad behaviour of the parturient which means either she practices adultery or if she behaves disrespectfully towards elders. In Dubréka, the respondents linked the occurrence of labour dystocia and obstetric fistula to witchcraft *koromikhi* in Sousou. In Labé, some respondents thought that obstructed labour and obstetric fistula are due to divine punishment in local dialect *lette Allah* when the woman does not respect her husband or has contracted the pregnancy out of the legal union. Others deemed that childbirth is difficult because of the narrowness of the delivery route in Fulani *lawol ngol no faadhi* in parturients who do not have sex during pregnancy or when the woman had not adequately had female circumcision, in local dialect *o suuwaaki laabhi* which means, part of the clitoris was left in place during the excision. The majority of respondents had a poor perception of health services (male staff, lack of privacy, poor hygiene, abuse). Several cultural practices (early wedlocks, genital mutilations, dietary restrictions, home deliveries) also induce obstetric fistulas.

**Conclusion:**

Respondents' cultural beliefs and practices regarding childbirth limit women's attendance at maternity hospitals during childbirth and favour obstetric fistulas.

## Introduction

Les fistules obstétricales (FO) sont des communications anormales entre le vagin et la vessie ou la vessie et le rectum ou le vagin et le rectum, causées par un accouchement prolongé et difficile en l'absence de soins obstétricaux appropriés [18, 23, 24, 26]. Elles provoquent une incontinence urinaire et/ou fécale chronique ayant des effets très préjudiciables sur la vie sociale et l'état de santé de la femme [3, 11, 14].

Les fistules génitales féminines surviennent généralement après un travail dystocique prolongé, chez des jeunes femmes présentant des séquelles d'excision ou des conséquences d'accouchements antérieurs dystociques entraînant une fuite continue et incontrôlée d'urines ou de fèces, entre autres séquelles débilitantes [3, 12, 34]. Ces fistules sont l'une des conséquences les plus graves d'un accouchement compliqué, car elles provoquent l'incontinence qui constitue une maladie humiliante qui relègue ces femmes en marge de la société, suscite des divorces et des répudiations.

On estime à environ 2 à 3,5 millions le nombre de femmes souffrant de manière permanente des problèmes liés aux FO dans les pays en développement, avec 50000 à 100000 nouveaux cas chaque année [27].

Pratiquement éradiquée dans les pays développés, les FO continuent à faire des ravages dans les pays pauvres notamment ceux de l'Asie et de l'Afrique subsaharienne [27, 33].

En Guinée, selon l'enquête démographique et de santé (EDS-IV 2012), la prévalence des FO varie selon les régions naturelles du pays. Cette prévalence est de 1,4% pour la Basse Guinée y compris Conakry la capitale, de 0,7% en Moyenne Guinée, de 0,8% en Haute Guinée et de 0,4% en Guinée forestière [19].

Selon la même source, 97% des femmes de 15-49 ans en Guinée sont excisées. Le type d'excision le plus pratiqué consiste en une section partielle du clitoris (84%): 6% des filles de moins de 15 ans qui ont été excisées ont subi une infibulation, c'est-à-dire une suture de la majeure partie des grandes lèvres de la vulve. Ces pratiques compromettent la vie sexuelle et reproductive des victimes et constituent un facteur de risque majeur des FO [19].

Les facteurs influençant l'utilisation des services de santé maternelle opèrent à différents niveaux: individu, ménage, communauté et état [4]. Ils ont été largement documentés dans la littérature et sont connus pour affecter l'utilisation des services de santé maternelle à travers le monde: éducation maternelle, âge de la femme, emploi et revenus, statut socio-économique, résidence (rurale / urbaine), parité, distance par rapport aux établissements de santé, exposition aux médias, accessibilité financière, dysfonctionnements de personnes, d'hospitalité et institutionnels, manque de confiance dans l'état, etc. [4, 6, 30, 32].

Au-delà de ces facteurs, le recours aux services de santé est très largement lié à des spécificités socioculturelles (croyances, pratiques culturelles, perceptions) des populations et à la qualité de la relation soignant-soigné [2, 7, 8, 9, 15, 20, 21, 25, 29, 31].

Les conséquences sanitaires, psychosociales et économiques des FO sont importantes pour les victimes, dramatiques pour toutes, mais en particulier pour les Musulmanes, considérées alors en état d'impureté permanente à cause de l'incontinence urinaire ou fécale et qui ne peuvent plus faire leur prière dans l'état de pureté rituelle requis par l'islam.

Au-delà des insuffisances structurelles du système de santé (manque de moyens, déficits et compétences des personnels…), les facteurs socio-anthropologiques sous-jacents qui contribuent à maintenir ce taux élevé sont peu explorés.

Il convient donc d'examiner les représentations des FO, les croyances et les pratiques des femmes en matière d'accouchement, d'analyser leur perception des services de santé.

## Patientes et Méthodologie

Cette étude a été réalisée dans trois préfectures de la Guinée qui ont été sélectionnées sur la base d'un choix raisonné en tenant compte des différences au point de vue ethnique et religieux entre les différentes régions du pays. Les régions choisies dans ce contexte sont:

- la préfecture de Kissidougou qui est située à près de 600 km de Conakry dans la région de la Guinée forestière. Cette préfecture est cosmopolite et on y trouve des Kissiens, des Malinkés, des Kourankos, des Lélés et d'autres ethnies venues d'autres régions du pays. On y rencontre des Musulmans, des Chrétiens et des Animistes;
- la préfecture de Labé située à 420 km de Conakry dans la région de la Moyenne Guinée qui est habitée par les Peuls pratiquant tous l'islam;
- la préfecture de Dubréka située à 50 km de Conakry dans la région de la basse Guinée habitée par les Soussous qui sont tous musulmans.

Dans ces trois sites, les pratiques de l'excision sont comparables en fréquence (98% selon EDS 2012) et sont surtout pratiquées par les exciseuses traditionnelles.

Dans la préfecture de Kissidougou qui compte 13 centres de santé (1 urbain et 12 ruraux), c'est le centre de santé urbain qui a été tiré au sort (avec 17 femmes). Dans la préfecture de Labé qui dispose de 14 centres de santé (3 urbain et 11 ruraux), le centre de santé rural de Sannou situé à 25 km de la ville de Labé a été tiré au sort (avec 13 femmes). Dans la préfecture de Dubréka, parmi les 11 centres de santé qui s'y trouvent (1 urbain et 10 ruraux), le centre de santé rural de Falessadè a été retenu suite au tirage au sort (avec 12 femmes).

Tous ces centres de santé disposent d'une maternité, d'une unité de consultations primaires curatives et d'une unité de vaccination et ils offrent tous des soins curatifs, préventifs et promotionnels. Le personnel est composé d'un médecin, d'une sage-femme et d'infirmiers.

Il s'agissait d'une étude qualitative d'une durée d'un mois allant de 15 janvier au 15 février 2018.

Cette étude a concerné les mères biologiques des nourrissons venues faire vacciner leurs enfants dans ces différentes structures, quel que soit leur lieu d'accouchement. Le choix a porté sur cette catégorie de personnes, car ce sont elles qui sont directement concernées par les FO et qu'elles ont une mémoire fraiche de leur accouchement.

Pour le recrutement des participantes à l'étude, nous avons réalisé un sondage de convenance, c'est-à-dire que les participantes ont été recrutées au fur et à mesure qu'elles se présentaient afin de faire vacciner leurs enfants. Deux enquêteurs (un gynécologue-obstétricien et une sociologue) ayant participé à d'autres enquêtes du même genre et parlant les langues locales de ces préfectures ont été recrutés et formés pour la réalisation de cette étude. Les entretiens ont été réalisés en français pour toutes les participantes scolarisées et en langues locales pour celles qui ne sont pas scolarisées (en malinké à Kissidougou, en soussou à Dubréka et en peul à Labé)

Pour la collecte des données, nous avons choisi l'entretien individuel afin de permettre aux participantes d'exprimer librement leur opinion.

Un guide d'entretien a été élaboré, pré-testé et validé auprès de femmes dans d'autres préfectures, différentes des trois sites de notre étude (cf. annexe).

En plus de l'enregistrement de l'entretien, une prise de note était faite en vue de relancer l'entretien ou pour faire préciser ou faciliter la mémorisation des points importants de l'entretien.

Au cours de la première rencontre, nous avons expliqué aux personnes retenues les objectifs de l'étude et leur avons présenté le formulaire de consentement éclairé pour obtenir leur accord.

Les conditions de l'entretien ont été négociées auprès de nos interlocutrices, quant à la date et la durée de l'entretien, le lieu (prioritairement à domicile ou dans les structures de santé) ainsi que l'absence de rémunération ou de compensation matérielle.

L'entretien débutait par les questions relatives aux caractéristiques sociodémographiques et se poursuivait sur: les représentations, l'image ou la compréhension que les femmes ont des FO; les croyances, l'opinion des femmes en matière d'accouchement heureux ou dystocique; les pratiques, les habitudes des populations en matière d'accouchement et de mariage; la perception, l'appréciation que les femmes ont des maternités.

La méthode de saturation a été utilisée au cours de cette étude afin de déterminer la taille de l'échantillon. On a considéré qu'il y avait saturation lorsque nous avons trouvé chez 4 à 5 personnes consécutives, des idées ou propositions déjà données par les prédécesseurs: nous avons considéré qu'il n'avait plus de nouvelles idées et l'enquête était clôturée.

La transcription de notes ou des entretiens enregistrés a été immédiatement réalisée par les enquêteurs après les entrevues. L'analyse a été faite selon les thématiques suivantes: les caractéristiques sociodémographiques, les représentations, les croyances, les pratiques et les perceptions.

## Éthique

Nous avons obtenu une autorisation des responsables des formations sanitaires. Le consentement libre et éclairé de toutes les répondantes a été également obtenu avant le démarrage de l'entretien semi-directif. Il leur a été expliqué, le but et le déroulement de l'étude, que la participation était volontaire, qu'elles étaient libres d'accepter ou de refuser l'entretien et que cela n'aurait aucune conséquence pour elles. L'anonymat et la confidentialité ont été respectés, c'est-à-dire que les noms des femmes n'étaient ni écrits ni enregistrés et que les notes prises et les enregistrements réalisés n'étaient détenus que par les enquêteurs et qu'il était convenu qu'ils ne seraient utilisés que pour les fins de cette présente étude. Tous les entretiens ont été enregistrés avec l'accord des répondantes.

## Résultats

### Caractéristiques sociodémographiques des femmes enquêtées

Onze femmes étaient des primipares, 9 des secondipares, 10 des paucipares et 12 des multipares; 9 femmes étaient âgées de moins de 18 ans, 25 avaient un âge compris entre 18 et 35 ans et 8 entre 36 et 40 ans; 28 femmes étaient non scolarisées en français et en arabe, contre 10 du niveau primaire, 4 du niveau secondaire. Parmi les 14 femmes scolarisées en français, 5 étaient scolarisées également en arabe. Les femmes mariées étaient majoritaires, 36, contre 6 célibataires; parmi les 36 femmes mariées, 15 appartenaient à un foyer monogame et 21 à un foyer polygame; les femmes au foyer étaient les plus nombreuses (17), suivies par les marchandes (10), les agricultrices (10) et les couturières (5).

Pour ce qui est du lieu du dernier accouchement, sur les 42 femmes, près de la moitié (19/42) avaient accouché à domicile.

Les représentations, les croyances et les pratiques culturelles en matière de fistules et d'accouchement sont à peu près identiques au niveau des différents sites enquêtés

### Représentations et croyances des répondantes en matière de fistules obstétricales

Certaines femmes (7/42) disaient qu'on parle de fistule obstétricale lorsque l'émission des urines échappe au contrôle de la femme, *bawlé dhen foutoto debbo* on en langue peule après un accouchement; 4 répondantes sur 42 définissaient la fistule comme une communication entre les voies urinaires et les voies génitales, en soussou k*ira na kholikira anoun dikira tagi* et en peul *debbo on yiiditi*. D'autres (9/42) disaient que c'est lorsque la femme ne garde pas les urines, *a mou kholi ragatama* en langue soussou. Un autre groupe (14/42) parlait de l'émission des urines par le vagin, en malinké s*ounadyi yebola a nyafe yoro lo*.

Les répondantes au niveau de tous les sites enquêtés considéraient les fistules comme des problèmes graves. La majorité des répondantes (30/42) pensaient qu'elles font suite à un accouchement chez une femme victime, soit de *gere* en peul ou de *korte* en peul, soussou et malinké, ce qui signifie mauvais sort, soit de *nyanne*, de *koro mikhi* en peul et soussou qui signifie sorcellerie; comme le disait une des répondantes: « …la femme dont je parle, elle n'était jamais d'accord avec sa belle-mère et tout le monde sait que c'est une grande sorcière. Voilà !»

Deux répondantes affirmaient que la fistule est causée par les djinns, *nyinne* en soussou; comme le précisait cette répondante, la fistule survient chez une femme qui habite sur le chemin des djinns qui se dit en soussou *nyinne kira*.

En revanche, 7 répondantes sur 42 estimaient que les FO sont liées à la volonté divine, en peul, *kodduruye Allah*, et les 3 autres pensaient que la fistule survient lorsque le bébé est mort dans le ventre de sa mère, en malinké *den bara sa konoro*.

Aucune femme n'a pu faire le lien entre le travail anormalement long et la survenue de FO.

### Croyances des répondantes en matière d'accouchement

Trois femmes sur les 42 pensaient que le travail d'accouchement se prolonge lorsque la femme a trompé son mari pendant la grossesse en ayant des rapports sexuels avec d'autres hommes, comme cette femme qui disait ceci: « …lorsqu'une femme a eu des rapports sexuels avec d'autres hommes pendant la grossesse, tant qu'elle ne dira pas le nom de tous les hommes avec lesquels elle a eu ces rapports, elle ne va pas accoucher ».

Sept femmes pensaient que c'est la sorcellerie qui empêche le travail d'accouchement de se dérouler normalement. Par exemple, l'une d'entre elle disait: « …si une sorcière ou une ennemie sait que tu es en travail, elle peut bloquer l'évolution du travail et t'empêcher d'accoucher tant qu'on ne t'a pas lavé avec des talismans » c'est-à-dire des remèdes liquides préparés par des guérisseurs traditionnels.

Deux femmes avançaient que le travail prolongé est lié au fait que la femme ne respecte pas son mari ou ses beaux-parents, comme celle-ci qui disait: « …une femme qui ne respecte pas son mari va souffrir au cours de l'accouchement et son enfant ne sera pas béni ».

Trois femmes pensaient que l'accouchement est difficile chez les femmes qui n'ont pas de rapports sexuels pendant la grossesse. L'une d'entre elles disait: « …ce sont les rapports sexuels pendant la grossesse qui facilitent l'ouverture du vagin et la sortie de l'enfant pendant l'accouchement ».

Sept femmes pensaient que le fait de ne pas exciser les femmes de façon propre, c'est-à-dire une excision complète du clitoris et des petites lèvres est une cause de travail prolongé. Une d'entre elles disait: « …si une femme n'est pas bien excisée, son accouchement va être compliqué et si l'enfant touche le clitoris au cours de l'accouchement, il sera aveugle ».

Trois femmes pensaient que le fait de consommer certains aliments pendant la grossesse peut rendre le travail difficile: « …si une femme consomme les oeufs pendant qu'elle est enceinte, son bébé va grossir beaucoup et son accouchement sera difficile et en plus le bébé sera un voleur ».

Trois femmes disaient que la présence d'hommes pendant qu'une femme est en travail va retarder l'accouchement et provoquer des problèmes chez la femme et son enfant. C'est ainsi qu'une d'entre elles soulignait: « …chez nous quand une femme est en travail, tous les hommes quittent le village et ne reviennent que quand la femme a fini d'accoucher quel que soit le temps que cela va prendre ».

Dix femmes sur les 42 pensaient que c'est le mauvais suivi prénatal qui retarde le travail d'accouchement: « …les femmes qui ne se font pas suivre pendant la grossesse souffrent toujours au cours de l'accouchement ».

Une autre femme rajoutait: « …moi je me suis bien fait suivre pendant ma grossesse et le Docteur m'a donné un médicament que j'ai pris à partir de 8 mois qui a facilité l'ouverture du col au cours de l'accouchement et j'ai accouché sans problème ».

En somme, les croyances en matière d'accouchement dans ces communautés ne favorisent pas les accouchements dans les structures de santé.

### Pratiques en matière d'accouchement et de mariage

Sept répondantes estimaient que l'accouchement est un phénomène naturel et que les femmes ne sont pas obligées d'aller accoucher dans une structure de santé s'il n'y a pas de problème. Par exemple, l'une d'entre elle affirmait: « …mes arrières-parents, mes parents ne sont pas nés dans une structure de santé et moi-même j'ai fait mes cinq accouchements sans problèmes à la maison avec l'aide de la vieille femme qui aide les femmes en travail dans le village ».

Une autre de renchérir: « …moi j'ai accouché seule mon dernier enfant, au champ, sans problème ».

Cinq femmes pensaient que le premier enfant doit naître dans la case familiale pour être protégé de la sorcellerie.

Une d'entre elles, pour illustrer cela disait: « …oui, oui, on avait dit à ma belle-soeur de ne pas aller accoucher au centre de santé et elle s'est entêtée avec son mari, mais le bébé n'a pas pu marcher jusqu'à l'âge de deux ans ».

Quatorze femmes déclaraient avoir fait tous leurs accouchements à la maternité et 5 disaient avoir accouché au moins une fois dans une structure de santé.

C'est le cas de cette femme qui disait: « …eh! moi je ne blague pas avec ça, dès que les douleurs commencent je pars à la maternité ».

Les pratiques culturelles en matière d'accouchement dans ces communautés ne sont pas en faveur de l'utilisation des maternités par les parturientes.

### Perception des services de santé par les répondantes

Sept femmes appréciaient bien les services de santé qui sont offerts dans les différentes structures et encourageaient les autres à y aller accoucher, comme cette femme qui faisait cet appel: « …je conseille à toutes les femmes d'aller accoucher au centre de santé, car on s'occupe bien des femmes maintenant et c'est gratuit ».

Douze femmes disaient que l'intimité des femmes est violée par la présence du personnel de santé de sexe masculin dans la salle d'accouchement et que cette pratique est contraire aux prescriptions de l'islam.

Une femme disait: « …moi ça me fait vraiment honte d'accoucher devant un homme ».

D'autres au contraire préféraient accoucher avec les hommes, car ils traitent mieux les femmes. C'est le cas de cette femme qui précisait: « …les hommes ne crient pas et n'insultent pas les femmes ».

Neuf femmes disaient que le personnel de santé ne respectait pas bien les femmes. Une d'entre elles soulignait: « …quand j'ai crié fort au moment de l'accouchement, la sage-femme m'a dit: est-ce que quand le bébé entrait, tu avais crié? Donc il ne faut pas nous tympaniser ».

Sept femmes disaient que le personnel de santé rançonnait les femmes après l'accouchement. Une parmi elles disait par exemple: « …on nous a dit que l'accouchement est gratuit, mais héé, on continue à réclamer de l'argent hein, ils ont toutes les stratégies ».

Une autre rajoutait: « …on m'a dit que c'est gratuit, mais on m'a exigé d'acheter certains médicaments pour accoucher vite sinon on allait m'opérer ».

Onze femmes disaient que la maternité était éloignée de leur village et que l'agent de santé était souvent absent: même si les femmes arrivaient jusqu'à la maternité, elles ne le trouvaient pas. C'est le jour du marché généralement qu'on le trouve. Une parmi elles a dit: « …ce n'est pas facile pour une femme en travail de marcher à pied pendant 2 à 3 heures de temps, surtout quand c'est pour aller trouver que le Docteur est absent ».

Trois femmes disaient que la césarienne était devenue une pratique trop fréquente dans les formations sanitaires. Une d'entre elles disait « …maintenant, dès que tu viens on te déchire le ventre », en langue peule *bhe seekete reedu ndun*.

Ces résultats mettent en relief la mauvaise image que les femmes ont des services de santé et qui a une influence négative sur la fréquentation des services de santé pour les accouchements.

## Discussion

La taille relativement faible de l'échantillon (42 femmes) n'a pas permis de mettre en évidence des différences régionales importantes au niveau des représentations, des croyances et des pratiques culturelles en matière d'accouchement malgré les différences ethniques, culturelles et religieuses entre les différents sites enquêtés.

### Représentations des fistules obstétricales par les répondantes

Parmi les répondantes qui déclarent connaître ce qu'est une fistule obstétricale et avoir connu dans leur entourage des femmes qui en étaient victimes, aucune n'a pu faire le lien entre sa survenue et le travail prolongé. Elles reconnaissaient toutes que ces femmes sont marginalisées, stigmatisées, souvent abandonnées par leurs maris et avaient peur de développer cette maladie. Elles savaient que c'est à la suite d'un accouchement que les fistules se produisent, mais elles pensaient que sa survenue était liée au mauvais sort jeté par la coépouse, la marâtre ou autres personnes ennemies ou bien qu'elle était liée au comportement de la femme, jugé mauvais selon les conventions sociales.

Un constat à peu près similaire a été fait en Éthiopien par Gebresilase [17] et au Cameroun par Tebeu PM [28], qui rapportent que leurs répondantes ne savaient pas que les fistules font suite à un accouchement difficile et prolongé. Elles pensaient plutôt que la survenue d'une fistule obstétricale était liée à une malédiction, à un mauvais esprit, à un mauvais comportement de la famille vis-à-vis de Dieu ou à une punition de Dieu.

Cette situation fait que lorsqu'il y a une dystocie, les femmes, au lieu de se rendre dans une formation sanitaire pour bénéficier d'une césarienne, ont recours plutôt aux marabouts, aux guérisseurs traditionnels etc. Et cela retarde leur prise en charge et favorise la survenue de FO.

### Croyances des répondantes en matière d'accouchement

Les résultats montrent, d'une manière générale, que les croyances en matière d'accouchement des répondantes limitent la fréquentation des maternités par les femmes au cours de l'accouchement. Le fait de croire par exemple qu'une femme en travail doit dire le nom de tous les hommes avec lesquels elle a eu des rapports sexuels pendant la grossesse pour qu'elle réussisse à accoucher fait penser que si elle a des difficultés au cours du travail, ce n'est parce qu'il y a une dystocie, mais c'est parce qu'elle cache plutôt les noms des hommes avec lesquels elle a eu des rapports: donc c'est sa faute. Ces croyances font que parfois, lorsqu'il y a une dystocie, au lieu de transporter rapidement la parturiente à la maternité pour qu'elle soit prise en charge, on pense que c'est un problème de mauvais comportement de la parturiente, de mauvais sort, de sorcellerie et on fait recours aux guérisseurs traditionnels (féticheurs, marabouts) qui utilisent soit des talismans, des herbes, de l'argile, des décoctions ou des incantations dans le but de faciliter l'accouchement. C'est après plusieurs jours de tentatives, au moment où généralement le bébé est déjà mort et la nécrose des tissus mous déjà produite, qu'elle accouche ou qu'elle est transportée à la maternité pour bénéficier d'une césarienne. Quelques jours après, la chute des escarres se produit et elle commence à perdre ses urines.

Ces croyances prédisposent aussi les femmes à d'autres problèmes de santé qui favorisent la survenue des dystocies. Lorsqu'on dit par exemple que si la femme n'est pas bien excisée, c'est-à-dire qu'on n'a pas enlevé totalement le clitoris, l'accouchement ne sera pas facile et que si l'enfant touche le clitoris de la mère au moment de l'accouchement, il sera aveugle, cela peut pousser les femmes à pratiquer des mutilations génitales très importantes ou à pratiquer même une deuxième excision, souvent avant le mariage, avec des risques de cicatrices vicieuses du périnée qui sont une source de dystocie et donc de fistule obstétricale. Les tabous alimentaires tel que le fait d'interdire la consommation des oeufs ou d'autres aliments (viande, pain) chez certaines communautés, kouranko et lélé à Kissidougou, pour ne pas que le bébé devienne voleur, prédispose les femmes à la malnutrition, à l'anémie, responsables de dystocie dynamique et de manque d'effort expulsif qui contribuent à prolonger le travail d'accouchement et à favoriser la survenue de FO.

Le fait aussi que les hommes quittent tous le village lorsqu'une femme est en travail limitent l'implication des maris dans la prise en charge des femmes et contribue à prolonger le travail dans la mesure où ce sont les maris qui autorisent la femme à aller à la maternité et ce sont eux aussi qui payent les frais de transports et d'accouchement.

L'ignorance par la population du mécanisme de survenue des FO fait que lorsqu'il a une dystocie on ne se dépêche pas d'envoyer la parturiente à la maternité pour qu'elle soit prise en charge avant la survenue de FO. Ce constat corrobore celui de Babalola au Nigéria qui rapportent qu'une minorité de femmes croit que les complications liées à l'accouchement sont causées par la sorcellerie: et les accoucheuses traditionnelles sont perçues comme mieux équipées pour intervenir dans ces cas [4].

### Pratiques en matière d'accouchement et de mariage

D'une manière générale, les pratiques qui ont été relevées au cours de cette étude ne sont pas non plus de nature à favoriser la fréquentation de maternités par les parturientes et augmentent le risque de dystocie.

En disant qu'une fille doit voir ses premières règles chez son mari, on favorise les mariages précoces de très jeunes filles et par conséquent les grossesses précoces avec leurs corolaires de travail d'accouchements difficile, prolongé, dystocique. À cet âge, les filles sont immatures et cette immaturité porte sur tout l'organisme y compris son bassin d'où la fréquence élevée des dystocies souvent notées chez ce groupe de femme. Si en plus on dit que le premier enfant, comme le rapporte Diallo en Guinée [13], doit naître dans la case familiale avec le risque élevé de disproportion, cela majore le risque de survenue de fistule obstétricale, car ce sont surtout les primipares qui sont concernées par les fistules. En cherchant à les faire accoucher en dehors des maternités, on empêche ou on retarde le diagnostic des dystocies qui sont responsables du travail anormalement long et donc de FO. Le fait également de se glorifier d'avoir accouché seule ou toujours à la maison encourage d'autres femmes à tenter d'accoucher à domicile, donc à ne se rendre dans les maternités que lorsque le travail a trop traîné, d'où un risque de fistule obstétricale. Ce même constat a été fait par Tsawe au Swaziland qui rapportent que le fait d'assister à des accouchements normaux à domicile dans la vie d'une femme ou de ses amis ou parents renforce l'idée que l'accouchement à domicile est sûr [32].

### Perception des services de santé par les répondantes

La perception que la plupart des répondantes ont des services de santé ne contribue pas à améliorer la fréquentation des maternités au cours de l'accouchement. La présence du personnel de sexe masculin dans les maternités fait que beaucoup de femmes à cause de leur croyance religieuse ne fréquentent pas les maternités et risquent d'accoucher sans une assistance qualifiée, avec toutes les conséquences que cela comporte sur la durée du travail. Le fait aussi de porter atteinte à l'honneur et à la dignité des parturientes, de les maltraiter ou de les rançonner, décourage les femmes à venir accoucher dans les structures de santé et les pousse à accoucher à domicile où les dystocies responsables des FO ne peuvent pas être identifiées et prises en charge, aggravant ainsi le risque de survenue d'une fistule obstétricale. Le mauvais traitement et le rançonnement ont été aussi évoqués comme facteurs qui limitent l'utilisation des services de santé par Babalola au Nigeria, Kujawski en Tanzanie, Abuya au Kenya et Baldé en Guinée [1, 4, 5, 22].

L'éloignement de certains villages par rapport aux structures de santé, surtout lorsqu'il est associé à un manque de moyens de déplacement, décourage les femmes à fréquenter les services de santé maternelle, surtout pour l'accouchement, comme l'ont rapporté aussi Tsawe au Swaziland, Cotter au Kenya, Gage au Mali et Diallo en Guinée [10, 13, 16, 32].

## Conclusion

Il a été noté au cours de cette étude que les femmes ignorent les mécanismes de survenue des FO. Aucune des répondantes n'a pu faire le lien entre accouchement dystocique et prolongé et la survenue de la FO.

Les croyances et les pratiques en matière d'accouchement ainsi que la mauvaise image que les femmes ont du système de santé à cause des nombreux dysfonctionnements qui le caractérisent limitent l'utilisation des services de santé maternelle, surtout pour les accouchements, et contribuent donc à la hausse de la prévalence des FO dans le pays.

## Conflits D'intérêts

Les auteurs ne déclarent aucun conflit d'intérêts.

## Annexe

### Guide D'entretien à Propos des Déterminants Socio-Anthropologiques de la Prévalence Élevée des Fistules Obstétricale en guinée

#### I- Identification de la Femme

1. Quel âge avez-vous?
2. Quelle est votre profession?
3. Quelle est votre situation matrimoniale?
4. Quel est votre régime matrimonial (monogamie ou polygamie)
5. Quelle est votre religion?
6. Quel est votre niveau d'instruction?
7. Combien de fois avez-vous accouché?
8. Où est ce qu'a eu lieu votre dernier accouchement?

#### II. Représentation de la Fistule Obstétricale

1. Avez-vous déjà entendu parler de la fistule obstétricale?
2. Si oui, selon vous qu'est-ce c'est?
3. Avez-vous souffert de la fistule obstétricale?
4. La fistule est-elle grave? Si oui en quoi?
5. Selon vous qu'est ce qui peut causer la fistule?
6. Peut-on l'éviter?
7. Connaissez-vous dans votre entourage une femme qui souffre de la fistule? Si oui, comment elle est perçue par son entourage?
8. Avez-vous autres choses à dire à propos de la fistule obstétricale?

#### III. Croyances en Matière D'accouchement

1. Selon vous combien de temps doit durer un travail d'accouchement?
2. A quoi peut-on attribuer les difficultés que les femmes rencontrent au cours du travail d'accouchement?
3. Qu'elles sont les conséquences d'un travail d'accouchement prolongé?

#### IV. Pratiques en Matière D'accouchement et de Mariage

1. Avez-vous accouché à domicile? Si oui combien de fois et pourquoi?
2. Selon vous qu'est ce qui peut amener les femmes à accoucher à domicile?
3. En cas de difficultés au cours de l'accouchement qu'avez-vous l'habitude de faire?
4. Quel est le rôle du mari et des beaux-parents dans l'accouchement?
5. À quel âge les filles doivent-elles se marier?
6. A quel âge vous-même vous vous êtes mariées?

#### V. Perception des Structures de Santé

1. Quelle appréciation faites-vous de la prise en charge des parturientes dans les maternités?
2. Êtes-vous satisfaites du personnel de santé?
3. Conseillerez-vous d'autres femmes à aller accoucher dans votre maternité?
4. Qu'est-ce que vous avez constaté dans les formations sanitaires qui peuvent décourager les femmes à venir y accoucher?
5. Avez-vous autres choses à dire sur les services de santé?
